# Effect of Food Containing Paramylon Derived from *Euglena gracilis* EOD-1 on Fatigue in Healthy Adults: A Randomized, Double-Blind, Placebo-Controlled, Parallel-Group Trial

**DOI:** 10.3390/nu12103098

**Published:** 2020-10-12

**Authors:** Takanori Kawano, Junko Naito, Machiko Nishioka, Norihisa Nishida, Madoka Takahashi, Shinichi Kashiwagi, Tomohiro Sugino, Yasuyoshi Watanabe

**Affiliations:** 1 Kobelco Eco-Solutions Co., Ltd., Kobe, Hyogo 651-2241, Japan; j.naito@kobelco-eco.co.jp (J.N.); m.nishioka@kobelco-eco.co.jp (M.N.); n.nishida@kobelco-eco.co.jp (N.N.); md.takahashi@kobelco-eco.co.jp (M.T.); 2 Soiken. Inc., Toyonaka, Osaka 560-0082, Japan; kashiwagi_shinichi@soiken.com (S.K.); sugino@soiken.com (T.S.); 3 RIKEN Center for Biosystems Dynamics Research, Kobe, Hyogo 650-0047, Japan

**Keywords:** *Euglena gracilis* EOD-1, β-glucan, paramylon, fatigue, selective attention, antioxidant

## Abstract

*Euglena gracilis* EOD-1, a kind of microalgae, is known to contain a high proportion of paramylon, a type of β-1,3-glucan. Paramylon derived from *E. gracilis* EOD-1 is presumed to suppress cellular oxidative injury and expected to reduce fatigue and fatigue sensation. Therefore, we aimed to examine whether food containing paramylon derived from *E. gracilis* EOD-1 (EOD-1PM) ingestion reduced fatigue and fatigue sensation in healthy adults. We conducted a randomized, double-blind, placebo-controlled, parallel-group comparison study in 66 healthy men and women who ingested a placebo or EOD-1PM daily for 4 weeks (daily life fatigue). Furthermore, at the examination days of 0 and 4 weeks, tolerance to fatigue load was evaluated using mental tasks (task-induced fatigue). We evaluated fatigue sensation using the Visual Analogue Scale, the work efficiency of the advanced trail making test and measured serum antioxidant markers. The EOD-1PM group showed significantly lower levels of physical and mental fatigue sensations and higher levels of work efficiency as well as serum biological antioxidant potential levels than the placebo group. These results indicate that EOD-1PM ingestion reduced fatigue and fatigue sensation, which may be due to an increase in antioxidant potential and maintenance of selective attention during work.

## 1. Introduction

Fatigue is a physiological alarm that reminds an organism of the need to rest to prevent exhaustion from overworking. Therefore, fatigue is a considerably familiar phenomenon in daily activities, such as work, exercise, creativity, and mental activity, and is essential for health maintenance.

Overall, fatigue is the difficulty in initiating or sustaining, voluntary activities [1]. In 2011, the Japanese Society of Fatigue Science defined fatigue and fatigue sensation as follows: Fatigue is a decline in the ability and efficiency of mental and/or physical activities that is caused by excessive mental or physical activities or disease. Fatigue is often accompanied by a peculiar sense of discomfort, a desire to rest, and a decline in motivation, which is referred to as fatigue sensation [2,3]. In other words, fatigue and fatigue sensation are distinguished. The subjective feeling of fatigue as a phenomenon that occurs in organisms can be said to be fatigue sensation. Furthermore, fatigue is classified into physical fatigue and mental fatigue. Physical fatigue, also known as peripheral fatigue, is caused by various muscle activities, while mental fatigue can be described as a state in which attention tasks that require self-motivation cannot be initiated or sustained in the absence of apparent cognitive impairment and deterioration of motor ability [1,4,5]. Moreover, overall fatigue, which is a combination of physical fatigue and mental fatigue, indicates the presence of overall fatigue sensation.

Fatigue can be quantified by various approaches (e.g., dysregulation of autonomic nervous system activity, hypothalamic-pituitary-adrenal axis hypofunction, immune abnormality, and reduction in work efficiency). In other words, fatigue is said to be due to reduced homeostasis (i.e., poor neural, endocrine, and immune interactions) caused by excessive activity and stress [1,3,6,7,8,9,10,11]. Fatigue has also been reported to be associated with oxidative stress [12] and reduced repair energy [13,14]. These associations are considered to be a few of the mechanisms of fatigue related to exercise and mental stress [15,16]. When an organism cannot process reactive oxygen species that are produced due to excessive activity, cell injury is induced and the immune cells that detect it generate cytokines, which serve as fatigue transmitters and inducers of fatigue sensation [9,17].

*Euglena gracilis* is a kind of microalgae that produces a β-1,3-glucan called paramylon. *E. gracilis* EOD-1 containing a high proportion (70–80%) of paramylon is known to proliferate depending on culture conditions [18,19]. Moreover, *E. gracilis* EOD-1, which has been suggested to have various functionalities, has recently drawn attention as a health food. Regarding its functionality, *E. gracilis* EOD-1 has been reported to improve health-related quality-of-life and immune function in humans [20]. There are numerous reports of the effects of paramylon derived from *E. gracilis* EOD-1 in diet-induced obese mice, such as the ability to prevent an increase in blood glucose levels during glucose load and reduce blood cholesterol and intraperitoneal fat weight [21]. In addition, paramylon has been shown to reduce cellular oxidative injury, improve liver disorder due to oxidative induction in rats, and increase reductase activity in the liver [22].

The abovementioned findings suggest that paramylon derived from *E. gracilis* EOD-1 reduces fatigue and fatigue sensation by maintaining immune function and reducing cellular oxidative injury. We have previously reported that food containing paramylon derived from *E. gracilis* EOD-1 (EOD-1PM) reduces physical fatigue sensation in daily life and increases pedometer steps and outing duration [23]. However, it has not been confirmed whether EOD-1PM actually reduces fatigue or not. Moreover, the mechanism of action has not been verified.

Against this backdrop, we conducted a randomized, double-blind, placebo-controlled, parallel-group trial in healthy individuals between the ages of 20 and 64 years to examine whether EOD-1PM reduces fatigue and fatigue sensation and to clarify the mechanism of action.

## 2. Materials and Methods

### 2.1. Subjects

Subjects were healthy men and women aged 20 to 64 years at the time of consent. The eligibility criteria were as follows: (1) those who had fatigue sensation in daily life, (2) those who agreed with the purpose of this study after receiving an explanation of the trial prior to participating, and (3) those who provided written informed consent. The exclusion criteria were the following 14 items: (1) those with severe cardiovascular disorders, liver dysfunction, renal dysfunction, respiratory disturbance, endocrine disorders, metabolism disorders, or those with a history of these conditions; (2) those with chronic fatigue syndrome or those who were deemed by the principal investigator to have severe fatigue, such as idiopathic chronic fatigue; (3) outpatients with chronic diseases; (4) those with mental illnesses such as depression/schizophrenia; (5) those with sleep disorders requiring treatment; (6) those who may have had an allergic reaction to the test food; (7) those who regularly used medical products and quasi-drugs with functional claims related to fatigue sensation that promote recovery from fatigue or nutritional supplements during physical fatigue; (8) those who regularly used Foods with Function Claims for reducing fatigue sensation; (9) heavy drinkers (those with pure alcohol consumption of 60 g/day or more); (10) those with a Body Mass Index (BMI) less than 17 or 30 or more; (11) those who provided more than 200 mL of blood within 1 month or 400 mL of blood within 3 months (donation, etc.) before the start of this trial; (12) those who participated in other clinical trials in the past 3 months or those who were currently participating in another clinical trial; (13) those who were pregnant or breastfeeding, or those who were planning to become pregnant; and (14) those who were deemed ineligible by the principal investigator. Sixty-six subjects who satisfied the abovementioned conditions were randomly assigned to two groups using random numbers by a staff member in charge of subject allocation (Statcom Co., Ltd., Tokyo, Japan) who did not participate in the trials directly. At the time of subject assignment, the staff in charge of subject allocation ensured that there were no significant differences in gender, age during the informed consent process, the score of overall fatigue sensation on the Visual Analogue Scale (VAS) [24], Pittsburgh Sleep Quality Index score [25,26,27], total power and low-frequency/high-frequency (LF/HF) of evaluation of autonomic nerve function [28], and the number of trials of the advanced trail making test (ATMT) [11]. The staff in charge of subject allocation sealed the allocation table immediately after the completion of subject allocation and safely stored it in a locked cabinet. The number of subjects was determined based on the results of similar trials that evaluated the anti-fatigue effects of food ingredients and compositions such as sesamin and astaxanthin [29], aged garlic extract [30], and lemon citric acid [31].

### 2.2. Test Food

Regarding the test food and placebo, EOD-1PM was used as the test food to be compared with the placebo. The dose of the *E. gracilis* EOD-1 (trade name: *Euglena gracilis* EOD-1, Kobelco Eco-Solutions Co., Ltd., Kobe, Japan) powder per capsule was adjusted so that the test food would contain 175 mg of paramylon derived from *E. gracilis* EOD-1, whereas the placebo contained dextrin. The test food and placebo were approved by the Institutional Review Board after passing an indistinguishability test.

### 2.3. Study Design and Intake Method

**Figure 1:**
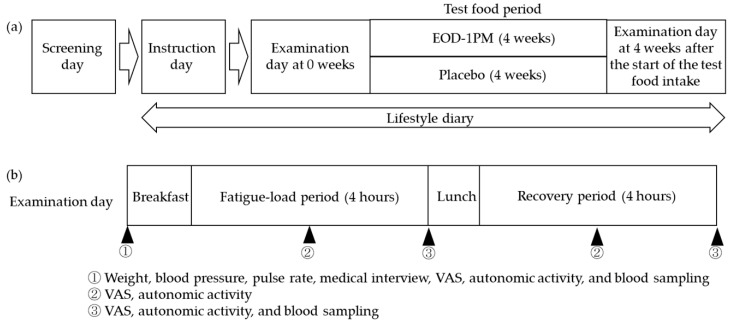
Study design and endpoints: (**a**) overall trial schedule, (**b**) examination schedule. EOD-1PM, food containing paramylon derived from *Euglena gracilis* EOD-1; VAS, Visual Analogue Scale.

This study was a randomized, double-blind, placebo-controlled, parallel-group trial. The study design is shown in Figure 1. The trial period consisted of the examination day at 0 weeks (ED0W), 4 weeks of test food intake, and the examination day at 4 weeks after the start of the intake (ED4W). A fatigue load was applied on the examination day, which was followed by a recovery period. An evaluation was performed in each period. Subjects were instructed to keep a diary from the instruction day to the ED4W. During the test food period, the subjects consumed two test foods or placebos once a day at dinner. During the trial period, subjects were not allowed to take drugs, quasi-drugs, and Foods with Function Claims related to fatigue sensation that promote recovery from fatigue or nutritional supplements during physical fatigue. In addition, they were instructed to maintain their normal lifestyle (e.g., alcohol, diet, exercise and smoking). On the day before the examination, the subjects had a designated dinner and were prohibited from consuming alcohol or caffeine-rich foods and beverages. On the day of the test, the subject had a designated breakfast and lunch. They were instructed to take only their designated food and water until the end of the test. This study was performed in accordance with the Declaration of Helsinki after the approval of the Institutional Review Board of Fukuda Clinic (approval number: IRB-20190216-5). The trial protocol was implemented after being registered in the University Hospital Medical Information Network Clinical Trials Registry (UMIN-CTR) (UMIN trials ID: UMIN000036314). This trial was carried out in Osaka, Japan.

### 2.4. Method of Fatigue Loading

Fatigue loading consisted of 4 sets (4 h) of 30-min 2-back tasks (simple working memory tasks) [4,32] and 30-min of the ATMT (selective attention and spatial working memory tasks) [4,11] (1 h in total). In the 2-back tasks, in which one letter of the alphabet was displayed every 3 s on the laptop screen, the subjects were asked to right-click the mouse if the letter matched the second-to-last letter, Otherwise, they were asked to left-click the mouse. In the ATMT (with three types of tasks, A, B, and C, in which 25 numbers from 1 to 25 were randomly presented on the laptop screen), subjects were instructed to left-click in order from 1 to 25. Tasks A, B, and C were repeated in order. In task A, the positions of 1 to 25 were fixed. Task B was the same as task A, except that when the subjects clicked the number, the number disappeared and a new number appeared at any position between 26 and 49. In task C, the arrangement of all numbers changed each time the target number was clicked. Regarding the fatigue loading, an instruction day was added to reduce the effect of the timing of fatigue loading.

### 2.5. Evaluation Method

The primary endpoint was the VAS [24] (overall fatigue sensation, physical fatigue sensation, mental fatigue sensation). Secondary endpoints were the evaluation of autonomic nerve function, work efficiency, blood oxidative stress, antioxidant markers, and safety.

Overall fatigue sensation, physical fatigue sensation, and mental fatigue sensation were evaluated by the VAS upon arrival to the facility, 2 and 4 h after fatigue loading, and 2 and 4 h after recovery on the ED0W and ED4W. The scores were evaluated according to the Guideline of Clinical Evaluation of Anti-fatigue by the Japanese Society of Fatigue Science [33]. Autonomic nerve function was evaluated by frequency analysis of the a-a wave intervals (maximum entropy method) by measuring accelerated plethysmography using ARTETT CDN (U-MEDICA, Inc. Co., Osaka, Japan) [28]. Measurements were taken upon arrival to the facility, 2 and 4 h after fatigue loading, and 2 and 4 h after recovery on the ED0W and ED4W. The endpoints were low-frequency component (LF, 0.04–0.15 Hz), high-frequency component (HF, 0.15–0.40 Hz), total power, and LF/HF.

Work efficiency was evaluated by the ATMT during fatigue loading on the ED0W and ED4W [4,11]. The endpoints [i.e., mean reaction time, standard deviation of reaction time, coefficient of variation (CV) of reaction time, and the number of errors in each task] were calculated as the total for the 1st to 4th sets.

Blood oxidative stress and antioxidant markers were evaluated during blood collection upon arrival to the facility, 4 h after fatigue loading, and 4 h after recovery on the ED0W and ED4W. The FREE CARRIO DUO system (Diacron International, Grosseto, Italy) was used to measure serum derivatives of reactive oxygen metabolites (d-ROMs) as an oxidative stress marker and biological antioxidant potential (BAP) as an antioxidant marker [34,35,36,37,38].

To evaluate safety, a blood test was performed upon arrival to the facility on the ED0W and ED4W. The endpoints were white blood cell count, differential leukocyte count (neutrophils, eosinophils, basophils, monocytes, lymphocytes), total protein, albumin, albumin/globulin ratio, aspartate aminotransferase, alanine aminotransferase, γ-glutamyl transpeptidase, creatinine, glucose, total cholesterol, high-density lipoprotein cholesterol, low-density lipoprotein cholesterol, triglyceride, creatine phosphokinase, uric acid, urea nitrogen, alkaline phosphatase, lactate dehydrogenase, Na, K, Cl, Ca, Mg, P, total bilirubin, and high-sensitivity C-reactive protein. The evaluation of the above endpoints was performed by BML, Inc. (Kawagoe, Japan), using a conventional method. An additional safety evaluation was conducted, and blood pressure, pulse rate, and body weight were measured, and medical interviews were performed upon arrival to the facility on the ED0W and ED4W. During the trial period, subjects were instructed to keep a diary on their lifestyle.

### 2.6. Statistical Analysis

For each endpoint, the values for each time point, changes before and after the intake of the test food, and changes from the time of facility arrival were evaluated using the following method. Regarding the intergroup comparisons, the Student’s *t*-test was used to analyze data with a normal distribution, whereas the Mann–Whitney U test was used to analyze data with a non-normal distribution. Regarding the intragroup changes in the values before and after the intake, a paired *t*-test was used since the data followed a normal distribution. For all analyses, the statistical software package IBM SPSS ver. 22.0 (IBM Japan Ltd., Tokyo, Japan) was used. *p* < 0.05 (two-tailed) was considered statistically significant. Values are presented as the mean ± standard deviation.

## 3. Results

### 3.1. Subjects

**Figure 2:**
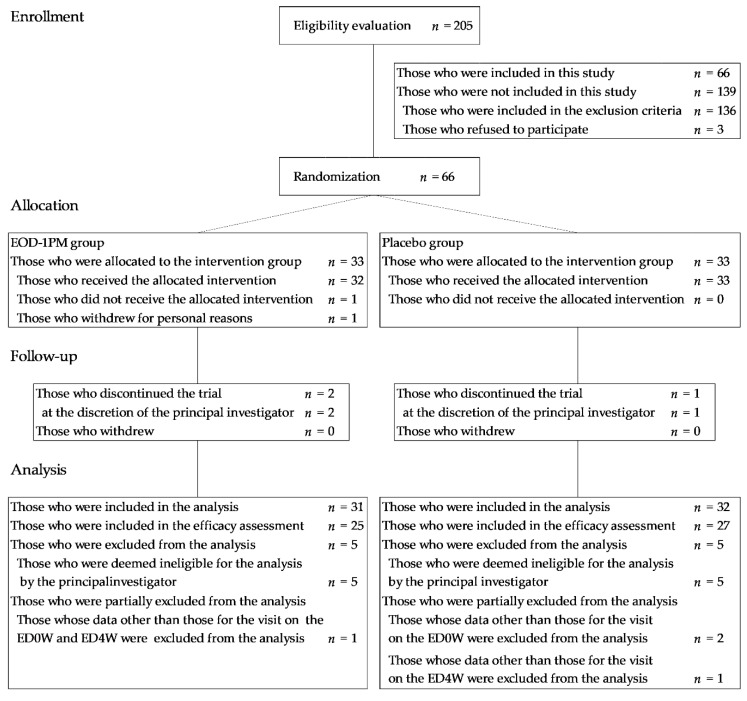
Flow diagram of participants. ED0W, examination day at 0 weeks; ED4W, examination day at 4 weeks after the start of the intake.

Figure 2 shows a flowchart depicting the process from the subject screening to the analysis. A total of 205 subject candidates provided informed consent and underwent screening. The screening for recruitment of healthy volunteers was done with the data of routine blood test and blood biochemistry test including the number of red blood cells and hemoglobin concentration in plasma. None of the volunteers entered in this study showed any abnormal values of them and other routine biochemistry data. They have no signs of anemia and other metabolic diseases. Sixty-six subjects, who were included in this study trial, were randomly assigned to two groups by a staff member in charge of subject allocation. The following three subjects were excluded from the analysis: (1) one subject who voluntarily withdrew from the trial for personal reasons, (2) one subject whose participation was discontinued at the discretion of the principal investigator after developing a chronic migraine during fatigue loading on the ED0W, and (3) one subject whose participation was discontinued at the discretion of the principal investigator after vomiting during fatigue loading on the ED0W (The subject did not receive the test food at all). A total of 63 subjects, after excluding the abovementioned three subjects, were included in the full analysis set for safety evaluation. Moreover, 52 subjects, after further excluding one subject whose participation was discontinued at the discretion of the principal investigator due to symptoms such as vertigo and nausea upon arrival to the facility on the ED4W and 10 subjects who were deemed by the principal investigator to be ineligible for analysis, were included in the per protocol set (PPS) for efficacy analysis. All the endpoint data after the start of fatigue loading for the following subjects were partially excluded from the analysis: three subjects who vomited during fatigue loading on the ED0W and two subjects who vomited during fatigue loading on the ED4W. In 62 patients, after excluding four patients who withdrew from this trial, the intake rate was as follows: 100% (*n* = 26) and 96.4% (*n* = 4) for the EOD-1PM group and 100% (*n* = 30) and 96.4% (*n* = 2) for the placebo group. Regarding the following subject backgrounds, no significant differences were found between the two groups for age, gender, BMI, VAS (overall fatigue sensation) at the time of the screening test, the Pittsburgh Sleep Quality Index, autonomic nerve function (LF/HF, total tower), and the number of ATMT trials at the time of instruction (Table 1 and Table 2).

### 3.2. Subjective Evaluation by the VAS

**Figure 3:**
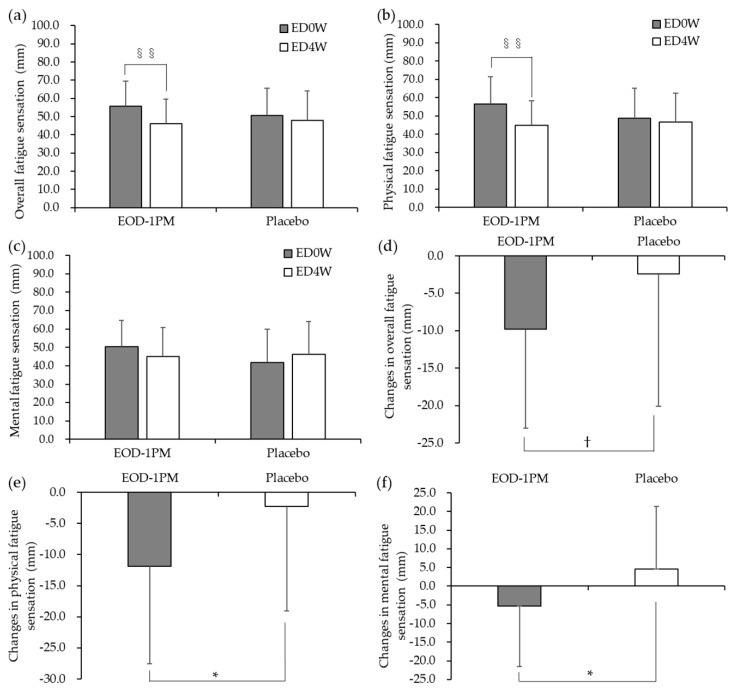
Evaluation of fatigue sensation by using VAS upon arrival to the facility on the examination day. (**a**) Comparison of overall fatigue sensation on the examination day at 0 weeks (ED0W) and at 4 weeks after the start of the intake (ED4W); (**b**) comparison of physical fatigue sensation on the ED0W and ED4W; (**c**) comparison of mental fatigue sensation on the ED0W and ED4W; (**d**) comparison of changes in overall fatigue sensation before and after the intake; (**e**) comparison of changes in physical fatigue sensation before and after the intake; and (**f**) comparison of changes in mental fatigue sensation before and after the intake. EOD-1PM group: *n* = 25, placebo group: *n* = 27. §§ *p* < 0.01. Comparison with that at facility arrival on the ED0W. * *p* < 0.05, † *p* < 0.1. Comparison between the placebo and EOD-1PM groups. The data are presented as the mean ± standard deviation.

Figure 3 shows the results of the evaluation of fatigue sensation by using VAS. The values of overall fatigue sensation and physical fatigue sensation upon arrival to the facility on the ED4W were significantly lower than on the ED0W, in the EOD-1PM group (*p* = 0.001 for both). On the other hand, no significant difference was observed in the placebo group (Figure 3a,b). Regarding changes before and after intake, physical fatigue sensation and mental fatigue sensation upon arrival to the facility were significantly lower in the EOD-1PM group than in the placebo group (*p* = 0.037 for both, Figure 3e,f). Overall fatigue sensation in the EOD-1PM group tended to be lower, but not statistically significantly more so than in the placebo group (*p* = 0.099) (Figure 3d).

### 3.3. Blood Cells and Blood Biochemistry Data

Statistically significant changes could not be observed before and after 4-week ingestion of EOD-1PM on blood cell counts especially the counts of leukocyte subpopulations and other routine blood biochemistry data indicating liver enzymes, kidney functions, and metabolic markers.

### 3.4. Oxidative Stress and Antioxidant Markers

In the evaluation of the serum d-ROMs values, BAP levels, and the BAP/d-ROMs ratio, BAP levels and the BAP/d-ROMs ratio upon arrival to the facility on the ED4W were significantly higher than those on the ED0W (*p* < 0.001, *p* = 0.014, respectively). On the other hand, no significant difference was observed in any of the items in the placebo group (Table 3). Furthermore, BAP levels and the BAP/d-ROMs ratio upon arrival to the facility on the ED4W were significantly higher in the EOD-1PM group than in the placebo group (*p* = 0.035, 0.01, respectively) (Table 3), and changes in BAP levels upon arrival to the facility before and after the intake period were significantly higher in the EOD-1PM group than in the placebo group (*p* = 0.043) (Table 3).

### 3.5. Evaluation of Fatigue Load and Work Efficiency

On the ED0W and ED4W, beyond the examinations of “daily-life fatigue” before fatigue-load tasks, we added the mental tasks with 2-back task and ATMT tasks (as shown in Figure 1). The overall, mental, and physical fatigue VAS scores were increased significantly after 4-h load (*p* < 0.01 for all scores before and after fatigue load), but the difference of the overall, mental, and physical fatigue VAS scores with fatigue load tolerance was not statistically significant. Such subjective scores were not altered statistically, but the objective markers of fatigue were changed as follows. In addition, we made the subtraction between the measurement values of each item at ED0W and ED4W and the subtracted values (Changes before and after intake of EOD-1PM or placebo) are also incorporated in the evaluation process as we had informed in the IRB protocol.

Table 4 shows the mean reaction time, the standard deviation of reaction time, the CV of reaction time, the number of errors of the total for all sets in the ATMT trials, and the changes before and after the intake for each ATMT task.

The comparison of values for the ED4W between the EOD-1PM and placebo groups showed the following results. In task A, the standard deviation and CV of reaction time were significantly lower in the EOD-1PM group than in the placebo group (*p* = 0.023 and 0.013, respectively). In task B, the standard deviation of reaction time was significantly lower in the EOD-1PM group than in the placebo group (*p* = 0.022). The CV of reaction time were also significantly lower in the EOD-1PM group than in the placebo group (*p* = 0.018). However, the CV of the reaction time on the ED0W were also significantly lower in the EOD-1PM group than in the placebo group (*p* = 0.029). In task C, the standard deviation and CV of reaction time were significantly lower in the EOD-1PM group than in the placebo group (*p* = 0.0066 and 0.0047, respectively).

Moreover, comparisons of the changes before and after the intake showed the following results: significantly lower CV of reaction time in task A, standard deviation and CV of reaction time, and number of errors in task C in the EOD-1PM group than in the placebo group (*p* = 0.018, 0.029, 0.020, and 0.044, respectively).

Table 5 shows the rate of correct responses in the 2-back tasks. No significant difference was observed for the ED0W and ED4W.

### 3.6. Evaluation of Autonomic Nerve Function

To evaluate LF, HF, total power, and LF/HF, frequency analysis of the a-a wave intervals of accelerated plethysmography (as an indicator of autonomic nerve function) was performed. In intergroup comparisons of values, a significant difference and similar tendency were observed for total power for both ED0W and ED4W. Furthermore, intergroup comparisons of the values showed no significant differences in LF, HF, and LF/HF.

Regarding the changes from the time of arrival to the facility (difference in pre- and post-intake), LF/HF at 2 h after recovery was significantly lower in the EOD-1PM group than in the placebo group (*p* = 0.031) (Table 6).

### 3.7. Safety Evaluation

There were no statistically significant differences in the results of physical examination between the EOD-1PM and placebo groups. Although the blood tests showed a slight statistically significant difference, since the values were within the standard values (the range of daily changes), the principal investigator determined that there were no clinically relevant changes. Furthermore, in this trial, no adverse events were attributed to the test food.

## 4. Discussion

This trial examined the effect of a 4-week intake of EOD-1PM on the reduction of fatigue and fatigue sensation in healthy adult men and women between the ages of 20 and 64 years.

The results showed a significant reduction in physical and mental fatigue sensation upon arrival to the facility before and after the 4-week intake in the EOD-1PM group compared to the placebo group. These changes may indicate that there was an effect on fatigue during the 4-week intake, suggesting that EOD-1PM reduced physical and mental fatigue sensation in daily life. In addition, the intra-group comparison showed a significant reduction in the values of overall fatigue sensation before and after the intake in the EOD-1PM group, whereas the placebo group showed no significant difference. Regarding changes before and after intake, overall fatigue sensation upon arrival to the facility tended to be lower in the EOD-1PM group than in the placebo group. The above results suggest that EOD-1PM also reduced overall fatigue sensation in daily life. In the previous study, we reported that four-week administration of EOD-1PM improves physical fatigue sensation in daily life [23]. The present study demonstrated the effect of EOD-1PM on the reduction of mental fatigue sensation and suggested that EOD-1PM was able to reduce overall fatigue sensation.

The relationship between fatigue and oxidative stress is well known. It has been reported that an oxidative stress marker, d-ROMs, and an antioxidant marker, BAP, are useful as biomarkers of fatigue [12]. The evaluation of the serum markers showed a significant increase in BAP and the BAP/d-ROMs ratio upon arrival to the facility on the ED4W of EOD-1PM and significant changes in BAP before and after the intake of EOD-1PM. On the other hand, no significant change was observed in d-ROMs. BAP, which measures the ability to reduce Fe^3+^ to Fe^2+^, is an index of antioxidant potential [35]. d-ROMs, which imply hydroperoxide levels, have been reported to be a useful marker of oxidative stress [34]. BAP, by its nature, may increase as oxidative stress increases. This study demonstrated an increase in antioxidant potential despite no increase in oxidative stress in the EOD-1PM group during the four-week study period. Therefore, EOD-1PM may reduce fatigue by permanently improving antioxidant potential without increasing oxidative stress. In addition, paramylon has been reported to increase reductase activities such as superoxide dismutase, catalase, and glutathione peroxidase in the rat liver [22]. EOD-1PM may also enhance these reductase activities and improve antioxidant potential. In most cases, studies in healthy subjects using food ingredients that have antioxidant effects show significant differences in the BAP/d-ROMs ratio [39,40]. Ingredients that change BAP alone are extremely rare. This is one of the characteristic properties of EOD-1PM. Therefore, EOD-1PM may have a considerably strong antioxidant effect.

It has been speculated that EOD-1PM is less susceptible to degradation by intestinal bacteria and is less likely to be absorbed through the intestines in mice [19,21]. Therefore, EOD-1PM may not be directly involved in improving serum antioxidant potential. Interestingly, EOD-1PM has been reported to induce the expression of genes, such as peroxisome proliferator-activated receptor-α, in the liver. EOD-1PM may be indirectly involved in gene expression in tissues by stimulating intestinal cells [21]. Regarding the findings of the effect of improving antioxidant potential in this trial, EOD-1PM may improve reductase activity through the same indirect action from the intestinal tract.

The evaluation of the results of the ATMT showed significant suppression of an increase in the standard deviation and CV of reaction time in tasks A and C and the standard deviation of reaction time in task B on the ED4W in the EOD-1PM compared to the placebo group. Regarding the changes before and after intake, the results also showed more significant suppression of an increase in the standard deviation of reaction time and CV of reaction time in tasks A and C and the number of errors in task C in the EOD-1PM group than in the placebo group. Therefore, it was found that EOD-1PM maintains work efficiency for a longer period of time by reducing the variation and changes in reaction time and the number of errors in the tasks. 

Fatigue is defined as a decline in the ability and efficiency of mental and/or physical activities [2,3]. In other words, maintenance of work efficiency indicates that fatigue is being suppressed [4,41,42,43]. There was no significant difference in the rate of correct responses in the 2-back tasks between the two groups, suggesting that the fatigue loading due to work was evenly distributed between the two groups and that EOD-1PM intake reduced fatigue due to workload. In addition, work efficiency in the ATMT confirmed in this trial was especially maintained in task C. Tasks A and B, in which the position of numbers does not change, assess both spatial working memory and selective attention as cognitive functions. On the other hand, task C, in which the position of numbers changes, assesses selective attention only (not spatial working memory) [4,11]. Because a marked effect that assessed selective attention was confirmed in task C, EOD-1PM may prevent the deterioration of selective attention at work, leading to the maintenance of work efficiency and the reduction of fatigue.

In the evaluation of autonomic nerve function (changes from the time of arrival to the facility [difference in pre- and post-intake]), LF/HF at 2 h after recovery was significantly lower in the EOD-1PM group than in the placebo group. It is known that LF mainly reflects sympathetic nerve activity, and HF reflects parasympathetic nerve activity [44], and that LF/HF, a relative indicator of sympathetic nerve activity, increases during fatigue [45]. EOD-1PM intake may expedite recovery from fatigue by promoting parasympathetic nerve activity early in the post-fatigue recovery period.

Moreover, it has been reported that the phenomenon of fatigue is closely related to immune dysfunction and that immunity decreases with stress and fatigue. A study of salivary secretory immunoglobulin A (sIgA), which is considered as one of the biomarkers of fatigue, showed a negative correlation between middle-age stress experiences and the secretion rate of salivary sIgA [46]. Furthermore, studies have shown that intense sports training and competition reduce the concentration and secretion rate of salivary sIgA [47,48]. In a previous study, we reported that daily intake (4 weeks) of *E. gracilis* EOD-1 increased both the salivary sIgA concentration and sIgA secretion rate [20]. *E. gracilis* EOD-1 may reduce fatigue by suppressing the reduction of immunity in fatigue.

This trial demonstrated that continuous intake of EOD-1PM reduces fatigue and fatigue sensation. The phenomenon of fatigue is said to be due to reduced homeostasis (i.e., poor neural, endocrine, and immune interactions) [1,3,6,7,8,9,10]. EOD-1PM has been found to recover parasympathetic nerve activity from fatigue and may also promote the maintenance of immune function. In other words, EOD-1PM may maintain homeostasis and reduce fatigue by affecting the autonomic nervous system and immune system. Fatigue is thought to be caused by oxidative injury due to reactive oxygen species produced by cell overactivity [9,17]. This trial suggested that EOD-1PM permanently increases antioxidant potential and reduces fatigue by reducing cellular oxidative injury. These results suggest that regular intake of EOD-1PM may lead to a fatigue-resistant body. Furthermore, the daily accumulation of fatigue is thought to lead to chronic fatigue [49]. EOD-1PM, which can reduce accumulation of fatigue, may have a preventive effect on chronic fatigue.

Regarding the safety, no adverse events were reported in this study from the volunteers who made the daily reports of ingestion, timing, and adverse events if any by the intake of EOD-1PM. Other trials of longer-term intake of EOD-1PM have also showed its safety [23,50]. In addition, the volunteers reported no stimulating and awakening effects of EOD-1PM unlike the intake of caffeine which causes an awakening effect sometimes confused with anti-fatigue effects.

This trial had several limitations. First, the sample size was limited. Second, the analysis was performed on the PPS. Third, this trial was considered to be within the range of daily fatigue. However, because the degree of fatigue could not be clearly shown, it was not possible to show how effective the EOD-1PM was in terms of fatigue levels. Fourth, this trial confirmed the effect of EOD-1PM by using long-term work on a personal computer as a fatigue load. However, the effect of EOD-1PM on fatigue due to high-intensity physical load, such as exercise, was unknown. Therefore, future studies should further explore these issues.

## 5. Conclusions

In this randomized, double-blind, placebo-controlled, parallel-group trial, a 4-week intake of EOD-1PM in healthy subjects was found to reduce physical and mental fatigue sensation in daily life. The results showed that EOD-1PM reduces fatigue, suggesting that EOD-1PM reduces fatigue sensation by preventing the daily accumulation of fatigue. The mechanism by which EOD-1PM reduces fatigue and fatigue sensation is believed to be by improving antioxidant potential, and sustaining selective attention.

## Figures and Tables

**Table 1:** Subject backgrounds.

	*n*	Men	Women	Age (Years)	BMI (kg/m^2^)
EOD-1PM	25	13	12	49.2 ± 6.5	23.4 ± 2.4
Placebo	27	12	15	48.9 ± 10.3	22.7 ± 2.6

The data are presented as the mean ± standard deviation. BMI, Body Mass Index; EOD-1PM, food containing paramylon derived from Euglena gracilis EOD-1.

**Table 2:** Subject backgrounds (test score and evaluated autonomic nerve function).

	Values at the Screening Test				Number of ATMT Trials at the Time of Instruction
	VAS (Overall Fatigue Sensation) Score	Pittsburgh Sleep Quality Index Score	Evaluated Autonomic Nerve Function (Total Power)	Evaluated Autonomic Nerve Function (LF/HF)	
EOD-1PM	59.4 ± 8.0	5.2 ± 1.9	1350.9 ± 961.9	1.202 ± 1.232	797 ± 128
Placebo	59.9 ± 7.2	5.1 ± 1.9	1447.2 ± 732.9	1.200 ± 1.253	803 ± 129

The data are presented as the mean ± standard deviation; VAS, Visual Analogue Scale; ATMT, advanced trail making test; LF/HF, low-frequency/high-frequency.

**Table 3:** The concentration of oxidative stress and antioxidant markers at facility arrival on the examination day.

		ED0W EOD-1PM (*n* = 25) Placebo (*n* = 27)	ED4W EOD-1PM (*n* = 25) Placebo (*n* = 27)			Changes before and after Intake EOD-1PM (*n* = 25) Placebo (*n* = 27)	
d-ROMs (U.CARR)	EOD-1PM	307 ± 58	309 ± 54			3 ± 21	
	Placebo	326 ± 41	329 ± 39			2 ± 31	
BAP (mmol/L)	EOD-1PM	2085 ± 131	2238 ± 130	*	§§	153 ± 171	*
	Placebo	2097 ± 190	2147 ± 168			50 ± 187	
BAP/d-ROMs	EOD-1PM	7.0 ± 1.4	7.4 ± 1.4	**	§	0.4 ± 0.8	
	Placebo	6.5 ± 0.9	6.6 ± 0.8			0.1 ± 0.9	

** *p* < 0.01, * *p* < 0.05. Comparison between the placebo and EOD-1PM groups. §§ *p* < 0.01, § *p* < 0.05. Comparison with that upon arrival to the facility on the examination day at the start of the intake. The data are presented as the mean ± standard deviation. d-ROMS, derivatives of reactive oxygen metabolites; BAP, biological antioxidant potential; ED0W, examination day at 0 weeks; ED4W, examination day at 4 weeks after the start of the intake.

**Table 4:** Evaluation of work efficiency by the ATMT.

		ED0W Total for All Sets EOD-1PM (*n* = 24) Placebo (*n* = 25)		ED4W Total for All Sets EOD-1PM (*n* = 24) Placebo (*n* = 26)		Changes before and after Intake Total for All Sets EOD-1PM (*n* = 24) Placebo (*n* = 24)	
**Task A**							
Mean reaction time (sec)	EOD-1PM	1.673 ± 0.532		1.836 ± 0.684		0.164 ± 0.241	
	Placebo	1.639 ± 0.337		1.993 ± 0.752		0.164 ± 0.208	
Standard deviation of reaction time (sec)	EOD-1PM	1.213 ± 0.457		1.445 ± 0.693	*	0.233 ± 0.382	
	Placebo	1.345 ± 0.492		2.053 ± 1.096		0.476 ± 0.535	
CV of reaction time	EOD-1PM	72.8 ± 19.8		78.6 ± 24.5	*	5.8 ± 11.3	*
	Placebo	80.8 ± 19.3		100.2 ± 33.3		17.9 ± 20.9	
Number of errors	EOD-1PM	25.0 ± 27.1		29.1 ± 32.7		4.0 ± 10.7	
	Placebo	35.0 ± 37.4		38.2 ± 38.5		4.5 ± 23.6	
**Task B**							
Mean reaction time (sec)	EOD-1PM	2.230 ± 0.616		2.392 ± 0.714		0.162 ± 0.242	
	Placebo	2.220 ± 0.465		2.693 ± 0.984		0.274 ± 0.446	
Standard deviation of reaction time (sec)	EOD-1PM	1.893 ± 0.554		2.183 ± 0.842	*	0.289 ± 0.530	
	Placebo	2.196 ± 0.751		3.034 ± 1.600		0.638 ± 1.261	
CV of reaction time	EOD-1PM	85.8 ± 16.8	*	91.2 ± 21.7	*	5.4 ± 12.7	
	Placebo	97.7 ± 20.0		109.6 ± 30.4		11.9 ± 23.2	
Number of errors	EOD-1PM	23.6 ± 27.2		31.0 ± 35.4		7.4 ± 13.4	
	Placebo	38.7 ± 36.0		41.4 ± 40.0		5.0 ± 17.9	
**Task C**							
Mean reaction time (sec)	EOD-1PM	3.303 ± 0.587		3.534 ± 0.735		0.231 ± 0.402	
	Placebo	3.341 ± 0.571		3.963 ± 1.047		0.390 ± 0.433	
Standard deviation of reaction time (sec)	EOD-1PM	2.099 ± 0.598		2.434 ± 0.904	**	0.335 ± 0.673	*
	Placebo	2.359 ± 0.773		3.502 ± 1.651		0.832 ± 0.840	
CV of reaction time	EOD-1PM	63.0 ± 11.3		67.8 ± 14.8	**	4.8 ± 9.3	*
	Placebo	69.5 ± 14.7		85.3 ± 25.6		13.7 ± 15.2	
Number of errors	EOD-1PM	31.0 ± 33.5		32.4 ± 38.4		1.5 ± 13.0	*
	Placebo	35.8 ± 36.5		44.8 ± 40.3		10.7 ± 17.6	

** *p* < 0.01, * *p* < 0.05. Comparison between the placebo and EOD-1PM groups. The data are presented as the mean ± standard deviation. ATMT, advanced trail making test; CV, coefficient of variation.

**Table 5:** The rate of correct responses in the 2-back tasks.

		ED0W Total for All Sets EOD-1PM (*n* = 24) Placebo (*n* = 25)	ED4W Total for All Sets EOD-1PM (*n* = 24) Placebo (*n* = 26)
Rate of correct responses	EOD-1PM	0.863 ± 0.102	0.853 ± 0.127
	Placebo	0.850 ± 0.093	0.841 ± 0.102

The data are presented as the mean ± standard deviation.

**Table 6:** Evaluation of autonomic nerve function: changes from the time of facility arrival (difference in pre- and post-intake).

		2 h after Load EOD-1PM (*n* = 24) Placebo (*n* = 24)	4 h after Load EOD-1PM (*n* = 24) Placebo (*n* = 24)	2 h after Recovery EOD-1PM (*n* = 24) Placebo (*n* = 24)		4 h after Recovery EOD-1PM (*n* = 24) Placebo (*n* = 24)
LF (msec^2^)	EOD-1PM	9.8 ± 373.5	41.9 ± 571.1	−34.8 ± 401.4		−88.1 ± 370.0
	Placebo	−112.7 ± 509.0	−89.1 ± 889.0	9.7 ± 512.9		−21.5 ± 675.2
HF (msec^2^)	EOD-1PM	98.0 ± 270.3	72.0 ± 426.6	183.7 ± 344.7		−50.0 ± 366.5
	Placebo	30.7 ± 405.0	50.1 ± 535.9	38.1 ± 365.4		−8.9 ± 583.1
Total Power (msec^2^)	EOD-1PM	469.9 ± 1780.1	460.9 ± 1548.5	421.2 ± 1281.5		−163.0 ± 1248.0
	Placebo	−60.0 ± 1435.1	154.4 ± 2270.9	121.4 ± 1967.4		437.0 ± 2888.1
LF/HF	EOD-1PM	−0.623 ± 1.903	−0.719 ± 1.555	−0.389 ± 1.024	*	−0.364 ± 1.187
	Placebo	−0.209 ± 1.913	−0.302 ± 2.379	0.463 ± 1.568		0.025 ± 1.400

* *p* < 0.05. Comparison between the placebo and EOD-1PM groups. The data are presented as the mean ± standard deviation. LF, low frequency component; HF, high frequency component.
